# PREDICTIVE VALUE OF NUTRITIONAL INDICES FOR SARCOPENIA TRANSITIONS IN MIDDLE-AGED AND OLDER ADULTS

**DOI:** 10.1093/geroni/igad104.2464

**Published:** 2023-12-21

## Abstract

Since malnutrition plays an important role in sarcopenia development, we aimed to investigate sarcopenia’s transitional dynamics and explore value of nutritional indices for predicting state transitions in middle-aged and older Asian adults. We analyzed data from 1910 participants in the WCHAT study who had complete information at baseline (2018) and at least one follow-up between 2019-2022. We defined the normal, sarcopenia, and intermediate subclinical states. Using a continuous-time multistate Markov model, we calculated transition probabilities and examined associations between nutritional indices and transitions. The probabilities of remaining stable versus progressing to the subclinical state in normal individuals were 53.4% versus 42.1% and 40.6% versus 49.0% at 2 and 4 years. In the subclinical population, their 2- and 4-year chances were 60.2% and 51.2% for keeping stable, 11.8% and 16.2% for developing sarcopenia, 28.0% and 32.6% for reverting to normal. For sarcopenic individuals, their likelihood of staying stable versus retrogressing to the subclinical state was 67.0% versus 26.3% and 48.3% versus 36.3% at 2 and 4 years. Increased albumin, BMI, calf circumference (CC), geriatric nutrition risk index (GNRI), mid-arm circumference (MAC), prealbumin, and triceps skinfold thickness (TST) was predictive for reversion to earlier states. Conversely, decreased BMI, CC, GNRI, MAC, and TST was associated with progression to sarcopenia. Vitamin D was not significantly associated with any transitions. This study reveals how sarcopenia changes over time and highlights usefulness of simple and cost-effective nutritional indices for predicting state transitions, which can help identify individuals at risk of sarcopenia and guide targeted interventions.

